# Completeness, consistency and non-duplicity of records of child sexual abuse on the Notifiable Health Conditions Information System in the state of Santa Catarina, Brazil, 2009-2019

**DOI:** 10.1590/S2237-96222022000100012

**Published:** 2022-07-11

**Authors:** Vanessa Borges Platt, Elza Berger Salema Coelho, Carolina Bolsoni, Doroteia Aparecida Höfelmann

**Affiliations:** 1 Universidade Federal de Santa Catarina, Programa de Pós-Graduação em Saúde Coletiva, Florianópolis, SC, Brazil; 2 Universidade Federal do Paraná, Departamento de Nutrição, Curitiba, PR, Brazil

**Keywords:** Sex Offenses, Childhood Sexual Abuse, Child Abuse, Public Health Surveillance, Abuse Notification, Cross-sectional Studies

## Abstract

**Objective:**

To evaluate the completeness, consistency and duplicity of records of child sexual abuse on the Notifiable Health Conditions Information System (SINAN) in Santa Catarina, Brazil, between 2009 and 2019.

**Methods:**

This was a descriptive and analytical cross-sectional study aimed to assess the quality of SINAN data regarding completeness, consistency and non-duplicity.

**Results:**

3,489 cases of violence were reported, with a 662.5% increase in the number of notifications in the period studied, with the increase in the number of referral centers for the care of people in situations of sexual violence in the state, explaining 46.7% of the variation in the number of cases, between the years studied. Consistency was excellent in 90.0% of the records; and completeness ranged between excellent and good in 92.3% of them. There was an increased trend in completeness for 14 variables in the period. There were no duplicate records.

**Conclusion:**

Data from the sexual violence against children surveillance system were considered adequate regarding the questions that were assessed in the study.

## INTRODUCTION

Child sexual abuse (CSA) is a complex phenomenon for several reasons, it happens in several ways and results from different relationships between family members, peer groups, institutions and communities. It occurs when a child is engaged in sexual activities that he or she cannot comprehend, for which he or she is developmentally unprepared and cannot give consent.^1^

Obtaining estimates of the prevalence of CSA is difficult, given the lack of conceptual, legislation and methodological uniformity, which implies high levels of underreporting. According to data from Dial 100, a channel for disseminating information on the rights of vulnerable groups and reporting human rights violations, created by the Brazilian government, 95,200 reports of violence against children and adolescents were registered in 2020.^2^ Of these, 14,621 were related to physical abuse, rape or sexual exploitation. It is worth highlighting that the perpetrator of abuse usually belonged to the same ethnic group and socioeconomic level as the victim.^3^

Tackling CSA is one of the Sustainable Development Goals (SDGs) for the 2016-2030 Agenda proposed by the World Health Organization (WHO) and included, among its targets to be achieved by 193 United Nations (UN) Member States, ‘ending abuse, exploitation, trafficking and all forms of violence against children’ by the end of a set period of time.^4^

The issue of violence in Brazil has received greater attention from both researchers and government institutions since the last three decades, resulting in the development of coping plans, whose epidemiological surveillance actions for violence were responsibility of the Ministry of Health.^5^ Thus, the Violence and Accident Surveillance System (VIVA), created by the Ministry of Health in 2006, began to record cases of violence and measure the magnitude of this serious public health problem.

**Table:** 

Study contributions	
**Main results**	There was a 662.5% increase in the number of notifications of sexual violence against children in Santa Catarina between 2009-2019. There were no duplicate records, consistency was excellent in 90.0% of the records, and completeness was considered good to excellent in 92.3% of them.
**Implications for services**	Data quality regarding the items evaluated, when quite adequate for making inferences, helps services and managers have a real notion of the information measured and subsidize the actions aimed to cope with the health condition.
**Perspectives**	This study aims to collaborate in order to corroborate the potential of SINAN as a surveillance tool for sexual violence against children, contributing to planning and evaluating public policies.

The VIVA system has integrated the Notifiable Health Conditions Information System (SINAN) since 2009,^6^ and in 2011, the notification of violence, in the health field, became compulsory for all services, whether public or private. In 2014, cases of sexual violence became the subject of immediate notification and communication to each municipal health department, within 24 hours after the victim had received care.^7^

The compulsory notification of cases of violence is a triggering action of procedures that help the application of immediate measures, aiming to break the cycle of violence and mobilize the child and adolescent protection network. Therefore, clear, complete and adequate epidemiological information is an essential source of data for planning, monitoring, implementing and assessing health actions, especially in countries and regions with wide socioeconomic inequality.^8^ A good quality database should be complete (with all diagnosed cases), consistent with the original data recorded in health care centers (reliability), without record duplicities, and their fields must be filled in properly.^9^

Thus, evaluating the quality of sexual violence data notified on SINAN can contribute to strengthening the surveillance system of this health condition. However, studies that analyze the quality of these data, especially aimed at violence, are still scarce.^8^ A recent literature review on the subject identified only one study evaluating the quality of records of sexual violence against women aged 10 years and over in Santa Catarina,^10^ but it did not find any studies that had analyzed the quality of records of child sexual abuse on the information system related to completeness, consistency and non-duplicity.

In this context, this study aimed to evaluate the quality of child sexual abuse database in Santa Catarina, precisely regarding the attributions of completeness, consistency and non-duplicity.

## METHODS

This was a descriptive and analytical cross-sectional study on SINAN/VIVA notifications of child sexual abuse (against children between 0 and under 10 years of age) in the state of Santa Catarina, in the period from 2009 to 2019. This age group corresponds to the WHO’s definition of ‘child’,^11^ also adopted by the VIVA system.^6^

The 2012 Demographic Census, conducted by Instituto Brasileiro de Geografia e Estatística (IBGE), classified Santa Catarina as the 20^th^Brazilian state in land area and the 11^th^in population size, with 7,164,788 inhabitants (2019), of whom 842,530 were children younger than 10 years old.^12^

Data from the Brazilian National Health System Information Technology Department (DATASUS) showed 1,585 health care centers/primary healthcare centers in the state of Santa Catarina in 2020.^13^ It is worth mentioning that CSA notification is compulsory on SINAN, in all those health care centers, and that, according to the National Health Establishment Registry (CNES), created in 2013, specialized services providing care for people in situations of sexual violence in Santa Catarina had 71 centers registered until December 2019.^13^

The following quality measurement attributes of a database were evaluated: consistency (of information), completeness (proportion of completed fields) and lack of duplicities.^14^

Consistency of an information system is defined as the proportion with which related variables present coherent, non-contradictory values,^6^ being classified into levels, according to the parameter adopted by Abath et al.:^17^ excellent (coherence levels equal to or greater than 90%), regular (from 70% to 89%) and low consistency (less than 70%). The percentage of inconsistency is calculated by dividing the number of notification forms with inconsistency in a given category (numerator) by the number of notification forms that contain the categories under analysis (denominator). Feasibility criterion for obtaining consistency data was decisive for the elimination of field variables such as ‘pregnancy in children under 10 years of age’. Incompatible variables that have changed over the years have also been eliminated.

Completeness attribute of a system is assessed by the number of records that have non-null values, and the fields considered incomplete are those filled as ignored or left blank. The analysis of this attribute was based on the Romero and Cunha score (2007), used by the Ministry of Health to estimate the degree of completeness of the variables, such as: excellent (equal to or greater than 95%), good (ranging between 90 and 94,9%), regular (ranging between 70% and 89,9%), poor (ranging between 50% and 69,9%) and very poor (less than 50%).^14^

In the linear regression analysis, the proportion of completeness of the variables was considered as a dependent variable (y), and the years of the period, as an independent variable (x). Regression analysis was performed using the Prais-Winsten estimator, together with the Cochrane-Orcutt method to correct serial autocorrelation.^18^

The annual percent change (APC) and 95% confidence intervals (95%CI) were calculated by adjusting the linear regression to the natural logarithm of proportions, adopting the year as a dependent variable.^19^ A reduction trend was considered when the 95%CI of annual percentage change were negative, an increasing trend when both were positive, and a stability trend when the confidence interval included both negative and positive values.

Regarding database completeness and consistency, we analyzed the variables with mandatory completion, considered by the Ministry of Health as important for the analysis of CSA and essential for epidemiological and operational analysis of case definition.^18^ All variables were analyzed regarding completeness and consistency for the years 2009 to 2019, calculating the percentage of complete fields and consistent combinations in each year.

The following variables were evaluated in relation to completeness: age, sex, race/skin color, schooling, presence of disability/disorder, municipality of residence, place of occurrence, occurrence of a repeated event, type of sexual violence, other sexual violence, sexual exploitation, pornography, rape, sexual harassment, relationship with the abused child (other ties, police, institutional, caregiver, acquaintance, brother, unknown person, child, stepfather, mother, father), number of aggressors, sex of the perpetrator, the perpetrator was drunk.

The variables used to verify consistency are presented in Box 1 .

**Box 1:** Variables used to check consistency

Age (< 10 years old)	*versus*	Schooling (five or more years of schooling)
Sexual abuse (yes)	*versus*	Type of sexual violence (“negative” for all types)
Pregnant	*versus*	Age (< 10 years)
Sex of the perpetrator of violence (male)	*versus*	Relationship (mother)
Disability/disorder (no)	*versus*	Any disability indicated
Number of individuals involved (one)	*versus*	Sex of the perpetrator of violence (both)
Age (< 10 years old)	*versus*	Work-related violence
Age (< 10 years old)	*versus*	Relationship (employer)
Sex (male)	*versus*	Pregnant
Sexual abuse (yes)	*versus*	Self-inflicted injury

Non-duplicity on SINAN was defined as a single degree of registration for each event (sexual abuse), which occurred with the same child. Therefore, duplication occurs when, among all records, the same event (with the same individual) has been notified more than once.^17^

The analysis was performed by exporting the report to Tabwin from the following SINAN variables: notification number, occurrence date, victim’s first/last name, date of birth, victim’s mother’s name, sex, violence notification date, notifying unit and identification of the health condition. The analysis was performed through the following combinations, comprised of distinct variables:

- Combination 1 = notification number + occurrence date + identification of the municipality + identification of the health condition + victim’s name.
- Combination 2 = victim’s name + notification date + identification of the unit + date of birth + victim’s mother’s name + notification number + date of occurrence + sex of the victim.

The analysis of any duplicate cases was performed on a case-by-case basis by means of manual verification. Once there was confirmed duplicate, we would remove it. The percentage of duplicate records considered acceptable was 5%, according to the parameter adopted by Abath et al.^17^ and Delziovo et al.^10^ This attribute is essential for the system, because repeated notifications overestimate the measure of disease occurrence (incidence and/or prevalence).^16^

The relationship between the number of notifications and the number of referral centers was analyzed using Spearman’s correlation method, and Pseudo-R2 for Poisson regression was used to quantify the percentage of determination of the number of centers over the number of notifications.

The analysis was performed using Stata, a statistical software (Stata College Station, USA), version 14.

The study project was approved by the Research Ethics Committee of Universidade Federal de Santa Catarina (CEPSH/UFSC), Opinion No.3,615,628, issued on October 10, 2019: Certificate of Submission for Ethical Appraisal (CAAE) No. 18203919.8.0000.0121.

## RESULTS

**Figure 1:**
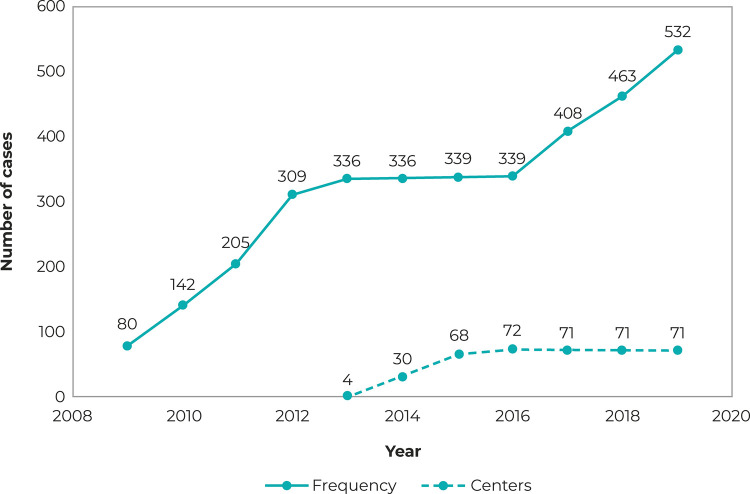
Distribution of the number of notifications of sexual violence against children (n = 3,489) on SINANa and number of health facilities specialized in sexual violence and registered with CNES, b state of Santa Catarina, 2009-2019

A total of 3,489 notifications of suspected or confirmed cases of child sexual abuse were made in Santa Catarina between January 2009 and December 2019. In that period, there was an increase in the number of notifications, and the number of referral centers, which increased from four in 2013 to 71 in 2019 ( Figure 1 ). There was a strong correlation (r = 0.89; p-value < 0.001) between the increase in the number of notifications and the number of referral centers, given that the increase in the number of centers has explained 46.7% of the variation in the number of cases over the years studied.

a) Sinan: Notifiable Health Conditions Information System; b) CNES: National Health Establishment Registry.

Duplicity was the first attribute of the quality of the information system that was evaluated. The analysis of the 3,489 notifications showed that there was no considerable number of duplicate records, thus, the quality of this item was considered acceptable (greater than 95%).

The percentage of consistency was excellent (greater than or equal to 90%) in nine out of the ten questions, and regular in one (between 70% and 89%). When the information related to the variables ‘under 10 years old’ and ‘five or more years of schooling’ was compared, only 13.4% of the records did not present consistency in relation to this information ( Table 1 ).

**Table 1:** Percentage of consistency and evaluation (E) of notifications of sexual violence against children, Santa Catarina state, 2009-2019

Check box fields	2009		2010		2011		2012		2013		2014		2015		2016		2017		2018		2019		Total		A
	n	%	n	%	n	%	n	%	n	%	n	%	n	%	n	%	n	%	n	%	n	%	n	%	
Age (< 10 years) *versus* schooling (≥5 years)	19/22	86.4	34/36	94.4	46/52	88.5	63/78	80.8	59/70	84.3	83/89	93.3	72/80	90.0	74/84	88.1	77/93	82.8	95/105	82.6	100/123	81.3	722	86.6	R
Sexual violence (yes) *versus* type of sexual violence (no)	67/80	83.8	125/142	88.0	182/205	88.8	282/309	91.3	303/336	90.2	303/336	90.2	314/339	92.6	311/339	91.7	373/408	91.4	430/463	92.9	497/532	93.4	3.187	90.4	E
Pregnant *versus* age (< 10 years)	80	100.0	142	100.0	205	100.0	309	100.0	336	100.0	336	100.0	339	100.0	339	100.0	408	100.0	463	100.0	532	100.0	3.489	100.0	E
Sex of the perpetrator of violence (male) *versus* bond (mother)	68	100.0	114/116	98.3	167	100.0	254/256	99.2	273/277	98.6	284	100.0	288/291	99.0	270/271	99.6	326/330	98.8	397/399	99.5	446/449	99.3	2.887	99.3	E
Disability/disorder (no) *versus* any disability indicated	73	100.0	126/127	99.2	183	100.0	278/281	98.9	310/312	99.4	315/318	99.1	311	100.0	324	100.0	391	100.0	421/424	99.3	487/488	99.8	3.219	99.6	E
Number of individuals involved (one) *versus* sex of the perpetrator of violence (both)	58	100.0	108	100.0	158	100.0	241/243	99.2	241/243	99.2	276/277	99.6	265/266	99.6	238/242	98.3	306	100.0	354/355	99.7	412	100.0	2.657	99.6	E
Age (< 10 years) *versus* work-related violence	78	100.0	139	100.0	202	99.5	307	100.0	332	100.0	332	100.0	336	100.0	339	100.0	408	100.0	460	100.0	528	99.8	3.461	99.9	E
Age (< 10 years) *versus* employer-bond	77	100.0	131	100.0	187	100.0	294	100.0	312	100.0	316	100.0	330	100.0	314	100.0	382	100.0	446	100.0	512/513	99.8	3.301	100.0	E
Male *versus* pregnant	25	100.0	46	100.0	56	100.0	102	100.0	102	100.0	100	100.0	90	100.0	82	100.0	104	100.0	112	100.0	113	100.0	932	100.0	E
Sexual violence (yes) *versus* self-inflicted injury	80	100.0	142	100.0	205	100.0	309	100.0	336	100.0	336	100.0	339	100.0	339	100.0	408	100.0	463	100.0	532	100.0	3.489	100.0	E

Legend: R = regular; E = excellent.

The completeness of seven variables was classified as excellent (percentage of filling in equal to or greater than 95%), good (percentage of filling in of the variable ranging between 90% and 94,9%) in 16, regular (percentage of filling in ranging between 70% and 89,9%) in two, and poor (percentage of filling in ranging between 50% and 69,9%) in a single variable. Taking into consideration all 26 variables, the proportion of completeness was 92.3%, which was considered good. The variable related to field 63 (‘suspicion of alcohol use by the perpetrator’) showed the lowest percentage of completeness: 68.1%.

Temporal trend of completeness of 14 variables presented an increase over the period, and the trend was statistically significant in 12 of them, excepting ‘age’, ‘sex’ and ‘municipality of residence’, to which the attribute analysis is not applicable. Trend of completeness in the nine remaining variables showed stability, corresponding to the following information: ‘schooling’, ‘presence of disability or disorder’, ‘place of occurrence’, ‘other sexual violence’, ‘pornography’, ‘rape’, ‘sexual exploitation’, ‘sex of the perpetrator’ and ‘the perpetrator was drunk’ ( Table 2 ).

**Table 2:** Percentage of completeness (C) and trend in the notifications of sexual violence against children, Santa Catarina state, 2009-2019

Completeness n (%)																
Check box fields	2009	2010	2011	2012	2013	2014	2015	2016	2017	2018	2019	TOTAL	C	Average annual percent change	Trend	p-value^a^
	80	142	205	309	336	336	339	339	408	463	552	3.489				
Age (< 10 years)	100.0	100.0	100.0	100.0	100.0	100.0	100.0	100.0	100.0	100.0	100.0	100.0	E	–	NA	
Sex	100.0	100.0	100.0	100.0	100.0	100.0	100.0	100.0	100.0	100.0	100.0	100.0	E	–	NA	
Municipality of residence	100.0	100.0	100.0	100.0	100.0	100.0	100.0	100.0	100.0	100.0	100.0	100.0	E	–	NA	
Race/skin color	96.3	96.5	92.2	94.5	93.2	98.5	96.8	97.6	98.8	98.1	97.2	96.3	E	1.17 (2.11;0.25)	A	0.038
Schooling	90.0	96.5	93.7	94.5	92.3	93.2	94.15	94.1	97.1	96.5	94.0	94.2	B	0.39 (-0.19;0.98)	E	0.289
Disability/disorder	97.5	96.5	95.1	96.2	97.0	96.7	94.7	96.8	98.3	94.4	95.7	96.3	E	-0.09 (-0.54;0.36)	E	0.745
Place of occurrence	95.0	93.7	85.9	91.3	92.9	92.3	91.5	92.9	93.9	92.9	93.6	92.3	B	0.94 (-0.10;2.00)	E	0.163
Repeat occurrence	63.8	70.4	73.2	75.1	70.8	74.4	74.6	78.2	76.0	78.4	76.7	73.8	R	2.29 (1.28;3.31)	A	0.005
Other type of sexual violence	76.3	78.9	84.9	87.7	94.4	86.9	89.4	89.4	91.2	91.4	91.9	87.5	R	1.92 (-0.49;4.40)	E	0.213
Pornography	85.0	90.9	84.4	89.3	96.7	90.2	90.9	89.4	91.4	91.6	94.0	90.3	B	1.13 (-0.05;2.33)	E	0.143
Rape	78.8	88.7	85.9	92.2	95.8	89.9	91.2	90.9	90.9	92.4	94.4	90.1	B	1.15 (-0.12;2.43)	E	0.162
Sexual harassment	83.8	90.1	85.9	93.9	95.8	90.8	94.1	92.6	92.9	93.7	94.4	91.6	B	1.38 (0.30;2.47)	A	0.061
Sexual exploitation	87.5	90.9	87.3	92.6	97.3	90.2	92.1	91.5	91.7	93.1	94.6	91.7	B	0.86 (-0.23;1.95)	E	0.215
Other bonds	86.3	83.1	88.3	93.5	91.7	92.6	95.6	92.3	92.2	95.3	94.7	91.4	B	2.30 (0.67;3.96)	A	0.043
Policeman	96.3	91.6	90.7	95.5	92.9	94.1	97.4	92.6	93.1	96.1	95.9	94.2	B	0.89 (0.23;1.55)	A	0.050
Institutional	97.5	95.1	97.6	97.4	98.5	97.6	100.0	100.0	100.0	100.0	100.0	98.5	E	1.14 (0.92;1.36)	A	0.001
Acquaintance	93.8	91.6	90.7	93.9	91.4	93.2	95.6	92.0	92.4	94.4	95.1	93.1	B	0.73 (0.24;1.23)	A	0.036
Brother	93.8	90.1	90.2	93.5	92.3	93.2	96.5	92.6	92.9	95.5	95.9	96.2	E	1.26 (0.70;1.2)	A	0.005
Unknown person	92.5	90.1	89.8	93.9	91.7	93.2	94.7	92.0	92.4	94.6	95.1	92.7	B	1.05 (0.55;1.56)	A	0.007
Son	96.3	92.3	91.7	94.8	93.2	94.1	97.4	92.9	93.6	96.3	96.4	94.4	B	0.88 (0.31;1.45)	A	0.029
Stepfather	93.8	91.6	90.7	94.5	92.0	94.1	95.9	92.3	93.1	95.5	95.5	93.5	B	0.89 (0.39;1.40)	A	0.016
Mother	93.8	91.6	90.7	94.5	92.0	93.2	95.6	92.6	92.7	94.2	95.3	93.3	B	0.70 (0.24;1.17)	A	0.032
Father	92.5	90.1	89.8	94.8	91.1	91.7	94.4	92.3	91.9	94.4	95.1	92.6	B	0.94 (0.43;1.45)	A	0.014
No. of perpetrators involved	87.5	91.6	87.8	91.2	85.7	90.8	92.6	90.3	91.2	91.4	91.7	90.2	B	0.72 (0.16;1.29)	A	0.061
Sex of the perpetrator	96.3	93.0	90.7	92.9	90.5	92.9	93.5	92.6	90.7	92.2	91.9	92.5	B	-0.06 (-0.58;0.47)	E	0.859
The perpetrator of violence was drunk	66.3	69.7	67.8	68.9	59.5	67.9	68.7	73.5	70.6	73.7	63.0	68.1	r	0.82 (-1.70;3.40)	E	0.600

a) P-value: p-value estimated using Prais-Winsten regression.

Legend: Completeness (C): E = excellent; B = good; R = regular; p = poor; Trend: NA = not applicable; I = increase; S = stability;

## DISCUSSION

This study showed a 662.5% increase in the number of notifications of child sexual abuse in Santa Catarina, between 2009 and 2019. Data quality related to the three attributes evaluated was considered high and therefore adequate for making inference. There were no duplicate records, and consistency was excellent in 90% of the variables, while completeness was good and/or excellent in 92.3% of them.

Temporal trend of completeness of 14 variables showed an increase over the period. The increase in the number of notifications, during the 11 years studied, can be justified by several factors, including the increase in the number of referral centers for the care of people in situations of sexual violence in Santa Catarina, all registered with the CNES as of 2013^13^ (a fact justified in 46.7% of the situations), as well as the real increase in the number of occurrences and greater awareness among professionals of the importance of their notifications, through the strengthening of sexual violence surveillance actions carried out by the state health services.^10^

The increase in the number of notifications of CSA may also result from activities developed by the State Department of Health, in partnership with the Ministry of Health and municipalities, through the decentralization of the SINAN system and conduction of training programs aimed at raising awareness and training of health professionals for the notification of violence,^10^ measures that other Brazilian studies have considered necessary and positive.^21^

In Pernambuco, between 2009 and 2012, there was a 212% increase in the number of notifications of violence against children,^17^ while in the state of Rio de Janeiro, in the period from 2009 to 2016, there was a 284% increase in the number of notifications of violence in all age groups.^20^ Based on data from SINAN, Veloso et al.^5^ found a 240% increase in the number of notifications of violence in Belém, capital city of the state of Pará, between 2009 and 2011, which according to the authors it resulted from the creation of new case notification centers in that capital. A similar hypothesis was raised by Delziovo et al.^10^ when evaluating notifications of sexual violence against women, in Santa Catarina.

The analysis of the attribute ‘non-duplicity’ showed an acceptable quality level, and it could be seen percentages lower than 5% of duplicate records, in agreement with other national studies that analyzed the quality of notifications of violence.^10^

Regarding the consistency of the system, in agreement with studies that evaluated the quality of SINAN data related to notifications of sexual violence against women in the state of Santa Catarina^10^ and self-inflicted or interpersonal violence in Recife, capital city of the state of Pernambuco,^17^ this analysis showed that the quality of the system was excellent in the state of Santa Catarina. The variables that presented a regular parameter of consistency among themselves were only those related to information on ‘under 10 years old’ and ‘five years or more years of schooling’. These results draw attention to the importance of training for the correct filling in of the notification form and better access to the instructional material for filling out notification forms, among health professionals.^23^ The material should be easy to consult, in addition to being kept up to date, taking into consideration that its latest issue dates from 2016.^6^ Moreover, children have started their school life earlier, and the professional responsible for filling out data may not be aware that a child under 10 years of age has no more than five years of schooling.

Filling out the CSA notification form on SINAN usually occurs while the victim is receiving care in hospital emergency services, usually overcrowded and with distinct and complex demands, which can affect the quality of the records made under these conditions. The emotional stress of a professional in charge of the care of the child and his/her family, which is usually weakened by the awareness of violence, and the need to comply with protocols in the different sectors responsible for this care, can also negatively interfere in the quality of filling out the notification form.^23^ In this context, the correct filling in of some fields of the form, such as those related to schooling, whose guidance is shown in the ‘Box of Equivalences between Teaching Nomenclatures’ of the instructional material,^14^ becomes unfeasible and/or unreasonable.

The attribute of completeness was classified as good and/or excellent in 92.3% of the records, a percentage higher than that found in a study conducted in Recife, in which the quality of notifications of interpersonal or self-inflicted violence was evaluated among all age groups,^16^ as well as in Santa Catarina, when evaluating the quality of notifications of violence against women, whose completeness was classified as good.^10^ Over the 11 years observed in this study, 14 out of the 23 variables analyzed for completeness showed a trend to improve the quality of filling out forms: a result considered quite positive, possibly attributed to the creation of more referral centers for the care of people in situations of sexual violence, the training of professionals and their greater familiarity with the notification form.

It could be seen that fields related to data about the perpetrator (sex, alcohol use), place of occurrence, typification of sexual violence, constitute or not sexual harassment or exploitation, schooling and presence of disability or disorder, showed stability in the quality of filling out a form. This stability trend in the completeness of some of the variables analyzed is possibly justified by age-related information biases, and its consequent ability to provide accurate information. Another factor that can contribute to information bias is the memory of the victim, taking into consideration that in most cases, the CSA is revealed after a long period of time has passed since the violence occurred,^25^ or even, due to the lack of sufficient discernment about the fact.

Regarding completeness, Rates and Mascarenhas^26^ suggested the hypothesis of information bias during data collection with parents or guardians, considering that in cases of CSA, most aggressors are part of the family environment and/or live with the children:^27^ sometimes it is the breadwinner, which may imply omission of data about the perpetrator, while they are filling out the notification form.

With regard to the stability of incompleteness of information related to the typification of sexual violence, such as ‘harassment’ and/or ‘sexual exploitation’, it may be related to the health professional’s lack of knowledge of the definitions of the event, or due to the professional’s lack of interest in correctly recording the events,^15^ or even because they consider filling out the notification forms to be a merely bureaucratic matter, without understanding the importance of data and information generation, either for (i the prevention and control of this type of violence, or for (ii service improvement^23^ aiming at conducting the case as a means of protecting the child.

A complicating factor in the adequate filling out of field 58 of Sinan form, related to the typification of sexual violence, is the use of legal terminology, such as ‘sexual harassment’ and ‘rape’, whose definitions are quite comprehensive. Using information on the degree of invasiveness of sexual violence, such as ‘violence with or without physical contact’, would be more appropriate, and in cases where there was a physical contact, specify whether or not penetration occurred – oral, anal or vaginal.^10^

It is also important to standardize definitions, terms and concepts used in the evaluation process, in order for the comparison of results between studies to be as comprehensive and better as possible.^15^

Frequent reviews on the quality of filling in the health information data are fundamental. Poor quality information can confuse the understanding of the epidemiological profile of the health condition, distort it, making it difficult to evaluate surveillance interventions.^25^

Taking into consideration territorial inequalities, especially with regard to technological resources available for the training of health professionals and managers aiming at the use of information, further studies, with systematic analyses that are adequate to the peculiarities of each state, are essential to reflect the real situation of the information system and CSA.^28^

According to Delziovo et al.,^10^ it is important to sensitize and instrumentalize health professionals, providing permanent education and return of the information generated from the data they had reported, in order to produce quality information, by improving the completion of the violence notification form on Sinan.^10^

The limitation of this study is the lack of filling in of all fields of the notification form (blank, missing and/or ignored), leading to different quantitative among the variables analyzed, a fact also observed by Canto and Nedel.^28^ Another limitation to be highlighted is the lack of stratification of the system analysis by municipality/health macro-region in Santa Catarina state; otherwise, it would be possible to detect local difficulties in filling in the notification form and, consequently, promote specific actions for each territory.

This study assessed in detail the quality of three attributes of SINAN in the notifications of CSA in the state of Santa Catarina. Taking into account the dimensions analyzed, the notifications of CSA in the period studied presented adequate percentages of non-duplicity, level of completeness ranging from good to excellent and excellent level of consistency in 90% of the topics evaluated, which have corroborated the reliability of the database for future inferences. The results obtained in this study, confirm the potential of the SINAN as a tool for CSA surveillance, aimed at planning and assessing public policies focused on the theme. They also contribute to raising awareness among managers, professionals, scholars and health teachers on the importance of adequate notification of these events, increased visibility and prevention of child sexual abuse in the state of Santa Catarina.
